# RETRACTION: Toxicity Evaluation of *Anacardium Occidentale*, the Potential Aphrodisiac Herb

**DOI:** 10.1155/bmri/9867162

**Published:** 2026-01-13

**Authors:** BioMed Research International

RETRACTION: J. Wattanathorn, P. Wannanon, S. Muchimapura, W. Thukham‐Mee, T. Tong‐Un, and P. Polyiam, “Toxicity Evaluation of Anacardium occidentale, the Potential Aphrodisiac Herb,” *BioMed Research Internationa*l, 2019, 1459141, https://doi.org/10.1155/2019/1459141.

The above article, published online on 21 February 2019 in Wiley Online Library (http://wileyonlinelibrary.com), has been retracted by agreement the journal Section Editor, Jinsong Ren, and John Wiley & Sons Ltd. The retraction has been agreed due to multiple instances of figure inconsistencies and duplications between figure panels.

The concerns were initially raised on PubPeer [1] regarding Figures 6 and 19. Subsequent investigation confirmed these issues, and identified further concerns with Figures 1, 2, 4, 5, 7, 8, 9, 10, 11, 14, 15, 16, 17, 18, 20 and 21. After assessment of the author’s response to the concerns, the editorial board find the results unrealiable and the article is retracted.

The authors did not respond to the retraction.

## References

[1] Actinopolyspora biskrensis , Toxicity Evaluation of Anacardium Occidentale, the Potential Aphrodisiac Herb, PubPeer. (2022) https://pubpeer.com/publications/C8569104C2938E4BE43988E5173C1A. 10.1155/2019/1459141 PMC6409010 30915346

